# Age Differences in Consumer Decision Making under Option Framing: From the Motivation Perspective

**DOI:** 10.3389/fpsyg.2016.01736

**Published:** 2016-11-07

**Authors:** Huamao Peng, Shiyong Xia, Fanglin Ruan, Bingyan Pu

**Affiliations:** Institute of Developmental Psychology, Beijing Normal UniversityBeijing, China

**Keywords:** emotion, information, age, decision, framing effects

## Abstract

Option framing effect is the phenomena that participants often accept more options when they are asked to delete undesired options from a full model (subtractive framing) than they do when they are instructed to add desired options to a base model (additive framing). Whether the same effect exists in different age groups is less well known. To explore the roles of age and purchase motivations on the option framing effect for automobiles purchases, this study adopted a 3 (age group: younger, middle-aged, vs. older) × 2 (option framing: additive vs. subtractive) × 2 (focus condition: information vs. emotion) mixed design. To manipulate purchase motivations, participants in the three age groups were instructed to focus on the ratio of utility and price of options (information-focus) or the extent of pleasure induced by the options (emotion-focus) when they made purchase decisions in two framing conditions. The results revealed similar option framing effect across all age groups in the information-focus condition regarding the total price paid for accepted options. In contrast, the framing effect was not found in the emotion-focus condition. In addition, older adults accepted more options and an overall higher price than younger and middle-aged adults in both focus conditions. This difference was more obvious in the emotion-focus condition than in the information-focus condition. Moreover, both the number of accepted options and the total accepted price of the younger group in the information-focus condition were higher than those in the emotion-focus condition, whereas the older and middle-aged groups accepted same number of options and price between two focus conditions. These results imply that purchase motivation is a moderator of the option framing effect and age characteristics linked with motivations must be considered in sales.

## Introduction

With the development of society and the economy, customers and their needs have grown increasingly diverse. Mass customization, which permits customers to select desired products and services, is considered to be the main mode of production (Gilmore and Pine, 1997; Da Silveira et al., 2001). Park et al. (2000) conducted research into automobile customization in order to investigate the framing effect. They employed “additive” and “subtractive” framing techniques to a series of decision tasks. The former framing presented customers with a base model and asked them to add the options they wanted. The more options added, the higher the price. The latter framing presented participants with a fully loaded product and asked them to delete options that they did not want. The price decreased as the number of deleted options increased. If all alternative options in the full model were deleted, the final price of the automobile equaled the price of the base model. The results showed that customers tended to choose more options with a higher total option price when they used subtractive rather than additive option framing, a finding that indicates the option framing effect. Levin et al. (2002) also found a similar framing effect in the purchase of pizza, a non-durable and less expensive item than an automobile. Framing effects have been found in automobile customization (Biswas and Grau, 2008; Goldstein et al., 2008; Biswas, 2009; Yang, 2010), condominium purchase (Pornpitakpan, 2009), mobile communication services (Jin et al., 2009), package tour services (Jin et al., 2012), and computer warranties (Dong, 2012).

Loss aversion, which depends on a reference point, is regarded as the main influential factor of the option framing effect (Kahneman and Tversky, 1979; Thaler, 1985; Johnson et al., 1993). The prospect theory (Kahneman and Tversky, 1979; Tversky and Kahneman, 1981) asserts that gains and losses are evaluated from a subjective reference point and individuals often weigh losses more than gains. When an alternative is used as a reference state or anchor, individuals will evaluate the losses from that state more than gains. For additive framing, a consumer reference point is the base model, whereas in subtractive framing, it is the fully loaded model. Hence, customers weigh gains and losses, based on different reference points. In additive framing, they need to assess the gains of adding option utilities and economic losses. In subtractive framing, they must weigh utility lost in deleting options and economic gains. Thus, customers are more likely to delete fewer products to avoid utility loss in subtractive framing, whereas, in additive framing, the customers are more likely to add fewer products to avoid economic loss.

Research on the option framing effect has mainly focused on younger adults (Park et al., 2000; Levin et al., 2002; Biswas and Grau, 2008; Biswas, 2009; Pornpitakpan, 2009; Jin et al., 2012). Whether the same effect exists for older adults is less well known. Research has shown that older and younger adults differ in their cognitive resources and behavior motivations (Schaie, 1994; Carstensen et al., 1999; Carstensen, 2006). Such age differences may affect the framing effects in different age groups.

Older adults have less cognitive resources than younger adults, which may lead to age differences in the option framing effect. It has been well established in the literature that cognitive abilities, such as processing speed, working memory, inhibition, episode memory, word fluency, spatial orientation, and reasoning decline linearly with increasing gage (Schaie, 1994; Salthouse, 1996, 2009; Baltes and Lindenberger, 1997; Shen et al., 2003; Anstey et al., 2012). This may affect the decision making of older adults. In addition, research has linked loss aversion to affective processes as opposed to deliberative processes (Biswas, 2009; Peters et al., 2011). Biswas (2009) found that customers had a larger option framing effect when making decision in an experiential mode and that the effect diminished when making decisions in a rational mode. The experiential/affective mode produces thoughts and feelings in a relatively effortless and spontaneous manner. The operations of this mode are typically rapid, automatic, intuitive, and appear to be based primarily on affective (emotional) feelings (Peters et al., 2011). If cognitive constraints become high, the option framing effect is shown to increase (Biswas and Grau, 2008). This phenomenon may arise because participants with greater cognitive constraints are more likely to use the experiential mode to make decisions. In contrast, when participants were asked to explain their choice (Miller and Fagley, 1991) or provide the rationale behind their selections (Sieck and Yates, 1997; Simon et al., 2004), framing effects were significantly reduced. The weakened cognitive resources of older adults may make them less dependent on controlled processing in decision making, which may lead to decision biases. For example, decreased processing speed and working memory can predict the lower decision quality of older adults (Henninger et al., 2010). Research has shown older adults tend to search less information and use simpler, less cognitively demanding strategies in their decision making (Mata et al., 2007). According to these findings, we may infer that older adults have larger option framing effects because of their reduced cognitive resources.

From the experimental mode perspective, emotional processing often leads to older adults performing more poorly in decision making tasks than younger adults. However, research based on socioemotional selectivity theory found that emotion played a different role in older adults’ decision making. Socioemotional selectivity theory (Carstensen et al., 1999; Carstensen, 2006) maintains that two broad categories of social goals shift in importance as a function of perceived time: those concerning the acquisition of knowledge and those concerning the regulation of stated emotions. When time is perceived as open-ended, goals focus on knowledge acquisition. This refers to acquisitive behavior geared toward learning about the social and physical world, including gathering information, experiencing novelty, and expanding knowledge. When time is perceived as constrained, the most salient goals tend to emphasize emotions and in particular, regulate emotional states. The delineation of two social goals concerns those who are primarily aimed at gaining knowledge or preparing for the future and those aimed at satisfying their emotional needs in the moment (Carstensen et al., 1999). Younger adults have wide time horizons and pursue goals related to knowledge. Older adults have restricted time horizons and therefore pursue goals related to emotion (Carstensen et al., 1999).

The difference in behavior motivations (social goals) may influence age differences in decision making. Fung and Carstensen (2003) found that in comparison to non-emotional advertisements, emotional advertisements were preferred and more readily remembered by older adults compared to younger adults. Löckenhoff and Carstensen (2007) investigated the differences between older and younger adults when making health care decisions. They found that older adults reviewed and recalled a greater proportion of positive rather than negative information compared to younger adults, when no extra instruction of motivation (control condition) existed. When motivational manipulations elicited information-gathering goals, these age differences were eliminated. In follow-up research (Löckenhoff and Carstensen, 2008) older adults exhibited a similar positive effect when they choose doctors for a social partner of a similar age. Moreover, different social goals also affected the decision quality of older adults. When they were instructed to focus on their feelings about health care choices (emotion-focus), their decision quality was better than when they focused on specific attributes and details (information-focus). In comparison, younger adults performed better in the information-focused condition than in the control condition (Mikels et al., 2010). In our study investigating a risky-choice framing effect, we found that in a high emotion arousal framing task (life-saving task), older adults did not exhibit framing effects while younger adults displayed the classic framing effect (Pu et al., unpublished). This is contrary to the assumption that older adults would show greater decision bias and poor decision quality because of their dependence on the experiential/affective processing mode.

In summary, we infer that decisions based on experiential mode would likely be different from decisions based on emotional goals. While decision making in experiential mode refers to how people process information, this conclusion does not suggest that the goal of decision making is to optimize emotions. Emotion goals based decision making does imply the aim of decision making, however, this process is not necessarily intuitive. It is our position that there will be a difference in the ultimate decision made based on the process of reaching that decision. Research has found that positivity effects based on emotional goals were only shown by older adults with access to greater cognitive resources (Mather and Knight, 2005). Decision-making based on emotional goals may therefore reflect controlled processing. Experiential mode requires fewer cognitive resources; thus, cognitive resource limitations make older decision makers more reliant on this processing mode. Processing based on emotional goals is not necessary affected by cognitive resources. Its decision goal is to optimize emotion, rather than to save cognitive resources. Though a link has been established between loss aversion and emotional reactions to losses and gains, it does not mean the direct purchase motivations of customers are to satisfy emotions. If a customer’s purpose is to satisfy emotions, the subjective value of losses and gains may not influence the purchase decision. Hence, in this study, customers’ purchase motivations are considered and age differences in the option framing effect connected with different purchase motivations will also be investigated.

The current study examined firstly any age differences for the option framing effect in the purchase of automobiles. Three age groups—younger, middle-aged, and older—were included, to enable an exploration of the option framing effect in the life-span context. From the loss aversion and experiential mode perspectives, it can be inferred that the option framing effect will increase as age increases, because cognitive resource attenuate with aging. However, the predicted age differences may be moderated by purchase motivations.

Secondly, we explored the role of purchase motivations and any interaction with age in the option framing effect. Two types of purchase motivations were examined. One is focused on information, which emphasizes the purchase decisions on the ratio of utility and price of options. The other motivation is focused on emotion, which emphasizes the purchase decisions on the extent of pleasure induced by options. For example, a customer may decide to buy a product only because it can produce a positive state of emotion regardless of utility and price. In the information-focus condition, the purchase motivation is connected to finding options with a high ratio of utility and price regardless of customers’ feeling, such as like or dislike of a particular option. Selecting or rejecting an option means a gain of utility/price or vice versa. Thus, loss aversion and reference point may affect the decisions of customers, which produce the option framing effect. It is hypothesized that older adults will exhibit a larger option framing effect than younger and middle-aged adults. Since middle-aged adults maintain comparative cognitive abilities in comparison to younger adults in longitudinal change (Schaie, 1994) they may have a similar framing effect to younger adults. To compare age differences for the option framing effect, analysis of variance (ANOVA) was utilized. A significant main effect of framing (additive vs. subtractive) would indicate that the option framing effect exists. An interaction effect of age × framing would indicate age differences exist within the option framing effect, namely, the option framing effect in the older age group would be larger than in the younger and middle-aged groups. In the emotion-focus condition, the extent of pleasure induced by options is explicit. Selecting or rejecting an option produces a gain of pleasure or a loss of displeasure, respectively. It is predicted there will be no trade-off between gains and losses. Therefore, loss aversion may not affect decision making and the option framing effect may not appear across three age groups.

In addition, we are also interesting in identifying which age group will spend more money on automobiles irrespective of option framing. The utility and price of options are stressed in the information-focus condition. Selecting or rejecting an option may represent an advantage of the automobile with high utility or not wasting money. In contrast, the emotion-focus condition emphasizes the emotion meaning of options. An option that represents a high level of pleasure may be expensive or not be useful in daily life, but it could make individuals to feel happy in the moment. Thus, the number of options and purchase amounts paid by the three age groups in the two alternate focus conditions may be different. The results of this problem may give us some suggestions in sale strategies from the perspective of age characteristics.

## Materials and Methods

### Participants

Three age groups, each with 60 healthy participants, were compared in this study. Sixty younger adults aged between 23 and 30 years (40 men and 20 women, *M* = 26.75, *SD* = 2.17), 60 middle-aged adults aged between 40 and 49 years (41 men and 19 women, *M* = 44.70, *SD* = 2.78), and 60 older adults between 60 and 83 years of age (30 men and 30 women *M* = 66.08, *SD* = 5.57) were recruited. The younger and middle-aged adults were company employees recruited through advertisements. The older adults were community residents recruited through advertisements. All participants were paid for 30CNY as compensation.

Participants in each age group were randomly assigned to one of two conditions: information-focus and emotion-focus. As shown in **Table 1**, there were significant differences in annual income [*F*(2,174) = 66.49, *p* < 0.001, $\eta_{p}^{2}$ = 0.433] and education level [*F*(2,174) = 65.07, *p* < 0.001, $\eta_{p}^{2}$ = 0.428] among the three age groups. The older group had significantly fewer years of education than the middle-aged and young groups. The difference between the middle-aged and young age groups was not significant on either variable. In addition, the older group had a significantly lower annual income than did the middle-aged and young groups. Although the annual income of the young group was slightly higher than older group, the difference was not significant. There was no significant differences of education years [*F*(2,174) = 0.67, *p* > 0.05, $\eta_{p}^{2}$ = 0.004] and annual income [*F*(2,174) = 2.20, *p* > 0.05, $\eta_{p}^{2}$ = 0.012] between two focus conditions regardless of age group. Thus, we included the years of education and annual income as covariates in our analysis.

**Table 1:** Descriptive statistics for age, education, and annual income by age group and focus condition.

Demographic variables	Older		Middle-aged		Young	
	Information focus	Emotion focus	Information focus	Emotion focus	Information focus	Emotion focus
	*M ± SD*	*M ± SD*	*M ± SD*	*M ± SD*	*M ± SD*	*M ± SD*
Age	67.10 ± 5.65	65.07 ± 5.38	44.97 ± 2.87	44.43 ± 2.71	26.47 ± 2.22	27.03 ± 2.11
Education (years)	12.97 ± 2.54	12.53 ± 2.70	15.50 ± 0.51	15.53 ± 0.51	15.97 ± .928	15.77 ± 1.01
Annual income (thousands CNY)	40.97 ± 19.51	42.07 ± 18.50	175.33 ± 107.50	146.67 ± 84.46	77.55 ± 29.74	66.00 ± 24.40

### Design

This study was a 3 (age group: young, middle-aged, vs. older) × 2 (option framing: additive vs. subtractive) × 2 (focus condition: information vs. emotion) design, with the age and focus conditions treated as between-subjects variables and option framing as a within-subjects variable. In previous studies investigating the option framing effect, option framing is typically designed as a between-subjects variable. Since individual differences may confound the effect of a between-subjects variable, option framing was designed as within-subjects variable in current study. This design also contributed to economic participation. As outlined in the Materials sub-section, in order to avoid participants making repeated decisions for same scenario, option framing was a within-subjects and between-materials design. The number and total price of the options accepted were included as the dependent variables. *The accepted options* in additive framing refer to those options participants added to the base model. The accepted options in subtractive framing refer to those remaining options after participant deductions from the full model. Thus, both accepted options in the two framing conditions represent a participant decision to buy.

The option framing manipulation asked participants to select a series of options that they would add to the base model and delete from the full model, according to the respective condition. Participants in the additive conditions viewed the base model and a list of options with their prices, which they could add. The full set of options added to base model represented the full model. Conversely, participants in the subtractive conditions viewed the full model and could delete options, such that deleting all the options would leave them with the base model.

The instructions were designed to temporally shift participants’ purchase motivations. Instructional manipulations in the emotion-focus condition asked participants to focus on their emotional reactions to the options and then make a choice. In the information-focus condition, participants were instructed to focus on the ratio of utility and price and then make a choice. The *emotion* refers to the level of pleasure induced by those options. The *utility* refers to the extent of perceived usefulness of those options. To ensure that participants focused on the instructed condition, they were asked to rate the ratio of utility and price or pleasure induced by adding or deleting options, depending on the focus condition, on a 5-point scale ranging from 1 (very low/very unpleasant) to 5 (very high/very pleasant) after each selection. This meant that participants in information-focus condition only rated the ratio of utility and price of the selected options and participants in the emotion-focus condition only rated the level of pleasure derived from the selected options.

The instructions for the two focus conditions were derived from research into older adults’ health care decision making (Löckenhoff and Carstensen, 2007; Mikels et al., 2010), which are as follows:

Information-focus condition: “If you want to buy this car, please only consider the ratio of utility and price of the options. The utility refers to the extent of usefulness of these options in your real driving. Which options will you choose?”
Emotion-focus condition: “If you want to buy this car, please only consider the degree of pleasure of the options. You can choose whatever option you like. Which options will you choose?”

### Materials

According to the two independent variables, there were four experimental materials, as showed in **Table 2**.

**Table 2:** Material allocations

Focusing condition Framing	Brand A (+OF) Brand B (-OF)	Brand A (-OF) Brand B (+OF)
Information focus	Material 1	Material 2
Emotion focus	Material 3	Material 4

*+OF is additive option framing. -OF is subtractive option framing.*

As previously stated, option framing was a within-subjects and between-materials design. Each participant accepted both additive and subtractive option framing, but the materials used in the two framings were different. For example, participant A accepted Brand A in additive framing and Brand B in subtractive framing (participant A would thus add options to the base model of Brand A and delete options from the full model of Brand B). Participant B accepted Brand A in subtractive framing and Brand B in additive framing (participant B would delete options from the full model of Brand A and add options to the base model of Brand B).

The two brands of cars each had seven options. The options of the two models differed so as to avoid the interference effect of repetitive options in the decisions of participants, but the prices of the options were same. To avoid the obvious different utility of seven options in each framing, which may lead to selection bias, ten auto experts were asked to rate the utility of each option on a 10-point scale. The non-parameter test showed no significant difference between all pairs of options. The utility values and prices of the seven options for the two models are presented in **Table 3**.

**Table 3:** Prices and utility scores of the two models’ seven options.

Number	Options of Brand A	Price (CNY)	Mean score of value	Options of Brand B	Price (CNY)	Mean score of value	*p*
(1)	Electric foldable outside mirrors	700	8.2	Outside rear-view mirror (dimming)	700	8.7	0.262
(2)	Adjustable headlamp (scope)	600	8.3	Headlight height adjustable	600	8.5	0.414
(3)	Outside mirror with heater	700	7.1	Outside rear-view mirror electric adjustment	700	7.8	0.059
(4)	Console screen (LCD)	800	7.7	ECU	800	8.3	0.262
(5)	Front parking radar	800	7.6	Reversing radar	800	8.5	0.197
(6)	Remote control key	600	7.9	Internally central door lock	600	7.9	1.000
(7)	LED taillights	600	8.4	Front fog lamp	600	8.7	0.518

All study material was originally presented in the Chinese language. Participants were provided with information about each option before they were asked to make choice. This served to counterbalance any effect from those with less experience with or knowledge of automobile products. The materials were presented to participants on a single page and included a description of the base/full model, followed by a list of the seven options to be added or deleted for each model.

### Procedure

Participants were asked to assume that they were facing the decision of purchasing a car and then instructed to read the relevant information in the questionnaire about the options available for the car. Before participants began to choose their preferred options, the instructions for the emotion-focus or information-focus conditions were read aloud to focus participants on different aspects of the alternative options when making their decision. As previously outlined, after each choice, participants rated the ratio of utility and price of each added or deleted option or the level of pleasure induced by the selected options.

This study was approved by the Ethics Committee of the School of Psychology at Beijing Normal University and written informed consent was obtained from all participants.

## Results

### The Extent of the Ratio of Utility and Price/Pleasure of Added/Deleted Options in Additive/Subtractive Option Framing

Repeated measures ANOVA was conducted on the scores from the ratio of utility and price (or pleasure) when participants focused on information (or emotion).As shown in **Table 4**, in the information-focus condition, the ratio of utility and price of added options (*M*_old_ = 3.85, *SD* = 0.67; *M*_middle_ = 4.43, *SD* = 0.39; *M*_young_ = 3.95, *SD* = 0.84) was higher than that of all deleted options in each age group (*M*_old_ = 2.41, *SD* = 0.96; *M*_middle_ = 2.55, *SD* = 0.47; *M*_young_ = 2.44, *SD* = 0.79], *F*(1,82) = 230.85, *p* < 0.001, $\eta_{p}^{2}$ = 0.737, and the rating scores in middle-aged group were higher than other two age groups [*F*(2,82) = 4.06, *p* < 0.05, $\eta_{p}^{2}$ = 0.09]. In the emotion-focus condition, the extent of the pleasure of the added options was higher than that of all deleted options in each age group, *F*(1,73) = 368.84, *p* < 0.001, $\eta_{p}^{2}$ = 0.835. There was no significant age difference in the rating scores. These results indicate that those options participants decided to buy were of a higher ratio of utility and price or a greater level of pleasure.

**Table 4:** The ratio of utility and price/pleasure evaluated by participants in additive and subtractive framing.

		Additive	Subtractive
		*M ± SD*	*M ± SD*
Information-focus	Older	3.85 ± 0.67	2.41 ± 0.96
(ratio of utility and price)
	Middle-aged	4.43 ± 0.39	2.55 ± 0.47
	Younger	3.95 ± 0.84	2.44 ± 0.79
Emotion-focus (pleasure)	Older	4.32 ± 0.48	2.41 ± 0.58
	Middle-aged	4.25 ± 0.32	2.80 ± 0.42
	Younger	4.28 ± 0.42	2.73 ± 0.70

### Number and Total Price of Accepted Options in Different Focus Conditions

Repeated measures ANOVAs, with the age grouping and focus conditions as between-subjects variables, option framing as a within-subjects variable, and years of education and annual income as covariates, were conducted. After controlling for education and annual income [accept number, *F*(1,172) = 0.004, *p* > 0.05, $\eta_{p}^{2}$ = 0.000; *F*(1,172) = 1.37, *p* > 0.05, $\eta_{p}^{2}$ = 0.008; accept price, *F*(1,172) = 0.02, *p* > 0.05, $\eta_{p}^{2}$ = 0.000; *F*(1,172) = 1.58, *p* > 0.05, $\eta_{p}^{2}$ = 0.009], option framing × age × focus condition interactions were not found for the number of accepted options [*F*(2,172) = 1.70, *p* > 0.05, $\eta_{p}^{2}$ = 0.19] or the total price paid for the options [*F*(2,172) = 2.08, *p* > 0.05, $\eta_{p}^{2}$ = 0.024]. The main effect of option framing and focus condition for the number and total price of chosen options was also non-significant. The descriptive statistics are presented in **Table 5**.

**Table 5:** Number and total price of accepted options in different focus conditions and option framings.

	Older		Middle-aged		Young	
	Information	Emotion	Information	Emotion	Information	Emotion
	*M ± SD*	*M ± SD*	*M ± SD*	*M ± SD*	*M ± SD*	*M ± SD*
**Number**						
Additive	4.67 ± 1.88	5.17 ± 1.74	3.57 ± 1.33	4.20 ± 1.77	3.90 ± 1.67	2.93 ± 1.51
Subtractive	5.00 ± 1.44	5.70 ± 1.42	4.57 ± 0.90	4.03 ± 1.61	4.67 ± 1.30	2.97 ± 1.87
3l**Price (CNY)**				
Additive	3170 ± 1298.32	3493.33 ± 1229.78	2466.67 ± 916.64	3183.33 ± 590.20	2690 ± 1154.11	1993.33 ± 1062.51
Subtractive	3426.67 ± 995.83	3890 ± 977.81	2943.33 ± 1193.86	2733.33 ± 1102.14	3253.33 ± 892.01	2063.33 ± 1277.25

A significant interaction of framing × focus condition on the total price paid for the options [*F*(1,172) = 3.96, *p* < 0.05, $\eta_{p}^{2}$ = 0.022] was found. For the number of accepted options, the interaction of framing × focus condition was not significant [*F*(1,172) = 3.33, *p* = 0.07, $\eta_{p}^{2}$ = 0.019] but showed a similar pattern to the total price.

**FIGURE 1:**
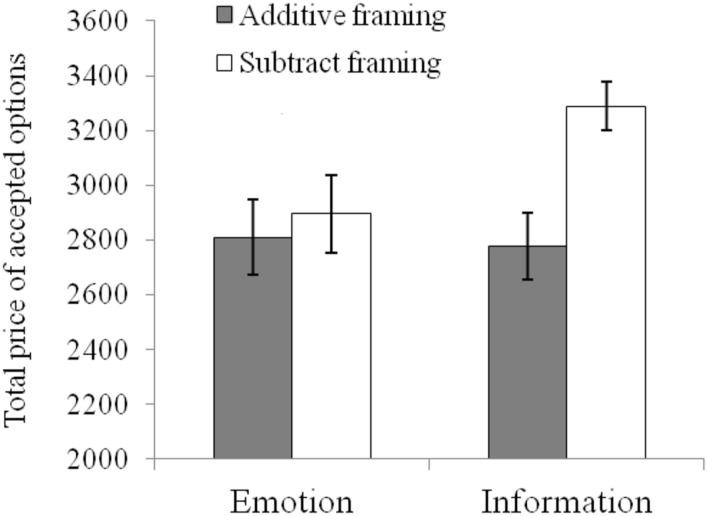
**The total price of participants’ accepted options in the two framing conditions**.

As shown in **Figure 1**, a simple effects test revealed the significant effect of option framing in the information-focus condition for the total price of the accepted options [*F*(1,172) = 12.42, *p* < 0.001, $\eta_{p}^{2}$ = 0.067]. The total price (3287.78 ± 840.52) in subtractive framing was higher than that in additive framing (2775.56 ± 1159.3). In the emotion-focus condition, the differences in the accepted price between the two framing conditions were not significant, *F*(1,172) = 0.49, *p* > 0.05, $\eta_{p}^{2}$ = 0.003. The results suggest that the option framing effect, as index by total price, appeared when participants focused on the ratio of utility and price. In contrast, the effect disappeared when participants focused on individual personal emotion.

The main effects of age for both number [*F*(2,172) = 16.97, *p* < 0.001, $\eta_{p}^{2}$ = 0.165] and the total price of the accepted options [*F*(2,172) = 15.36, *p* < 0.001, $\eta_{p}^{2}$ = 0.152] were significant. The number of options (10.27 ± 2.62) and total price (6990 ± 1812.36) of the older group was significantly higher (*ts* < 0.01) than those of the middle-aged group (8.18 ± 2.14; 5663.33 ± 1446.32) and the younger age group (7.23 ± 2.91; 5000 ± 2012.33). The difference between the middle-aged and younger groups was not significant (*t* > 0.05). The interaction of age × framing failed to be found [accepted number, *F*(2,172) = 0.28, *p* > 0.05, $\eta_{p}^{2}$ = 0.003]; accepted price, [*F*(2,172) = 0.532, *p* > 0.05, $\eta_{p}^{2}$ = 0.006], which indicated that the age differences were the same regardless of additive or subtractive framing. However, a significant interaction of age × focus condition was found for both the number of accepted options [*F*(2,164) = 9.94, *p* < 0.01, $\eta_{p}^{2}$ = 0.104] and the total price [*F*(2,172) = 10.040, *p* < 0.01, $\eta_{p}^{2}$ = 0.105]. Simple effects tests showed that the age differences arising from the number [*F*(2,172) = 24.36*, p* < 0.01, $\eta_{p}^{2}$ = 0.361] and total price [*F*(2,172) = 23.12*, p* < 0.01, $\eta_{p}^{2}$ = 0.212], in the emotion-focus condition were greater than those in the information-focus condition [accepted number, *F*(2,172) = 5.17, *p* < 0.01, $\eta_{p}^{2}$ = 0.057; accepted price, *F*(2,172) = 4.39, *p* < 0.05, $\eta_{p}^{2}$ = 0.049].

**FIGURE 2:**
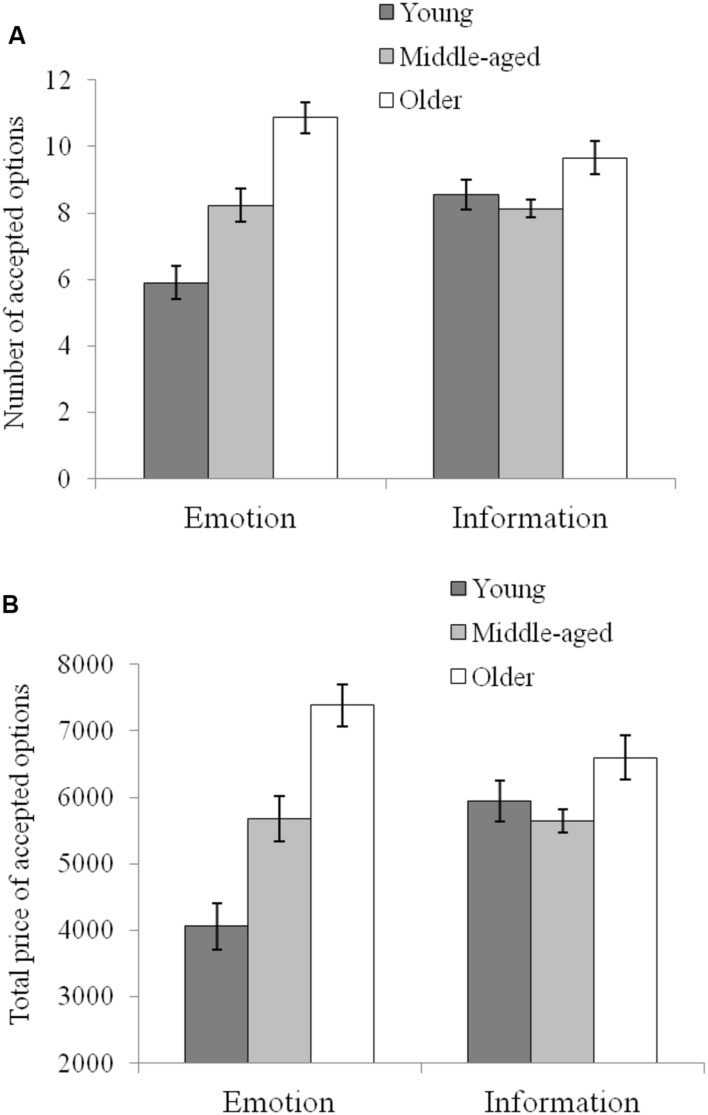
**(A)** Displays the number of accepted options for each age group across the different focus conditions. **(B)** Displays the total price of accepted options for each age group across the different focus conditions.

Moreover, as shown in **Figure 2**, simple effects tests of age × focus condition revealed that, in the younger age group, the number of options (8.57 ± 2.44) and total price (5943.33 ± 1678.81) in the information-focus condition were higher than the number (5.9 ± 2.75) and total price (4056.67 ± 1891.85) in the emotion-focus condition [accepted number, *F*(1,172) = 17.01, *p* < 0.01, $\eta_{p}^{2}$ = 0.090; accepted price, *F*(1,172) = 18.04, *p* < 0.001, $\eta_{p}^{2}$ = 0.095]. This result demonstrates that young adults are more willing to spend money for the ratio of utility and price rather than for emotional satisfaction. For older adults, the number and total price in the emotion-focus condition were slightly higher than those in the information-focus condition, but the differences were not statistically significant [accepted number, *F*(1,172) = 3.51, *p* = 0.063, $\eta_{p}^{2}$ = 0.020; accepted price, *F*(1,172) = 3.19, *p* = 0.076, $\eta_{p}^{2}$ = 0.018]. For the middle-aged adults, there were no differences in the number of options and the price between two focus conditions [accepted number, *F*(1,172) = 0.27, *p* > 0.05, $\eta_{p}^{2}$ = 0.002; accepted price, *F*(1,172) = 0.17, *p* > 0.05, $\eta_{p}^{2}$ = 0.001].

## Discussion

This study explored the option framing effect of automobile purchases in different focus conditions. The existence of age differences within the framing effects was also examined. The results showed that the option framing effect regarding the total price paid for the accepted options was significant in the information-focus condition. The index for the number of accepted options showed a similar pattern but did not reach statistical significance. Participants accepted higher prices in subtractive framing than they did in additive framing. No age difference for the framing effect was found in the information-focus condition. When participants made decisions based on their feelings about the options, the framing effect did not occur in any of the age groups, irrespective of the accepted number or price. In addition, the number of options and the price accepted by older adults were higher than those accepted by young and middle-aged adults, and this difference was more obvious in the emotion-focus condition than in the information-focus condition. Moreover, the number of accepted options and total accepted price of the younger age group in the information-focus condition were higher than those in the emotion-focus condition, whereas the older accepted a slightly higher number of options and price in the emotion-focus condition than in information-focus condition, although the differences were not significant. The middle-aged adults accepted same number of options and price in the two focus conditions.

### Purchase Motivation Is One of the Moderators of the Option Framing Effect

Though considerable research in psychology (Shafir, 1993), organizational behavior (Huber et al., 1987), and marketing (Biswas and Grau, 2008) has testified to the different roles of additive and subtractive framings in many situation (customers tended to accept more products and higher prices in subtractive framing), moderators of the option framing effect have been identified. Product categories, prices, anticipated regrets (Park et al., 2000), culture differences (Levin et al., 2002), and cognitive constraints (Biswas and Grau, 2008) have been shown to influence the direction and effect size of framing. The results of our study reveal the existence of a significant option framing effect in the information-focus condition, which is consistent with the findings of previous research (Park et al., 2000; Levin et al., 2002; Biswas and Grau, 2008; Goldstein et al., 2008; Biswas, 2009; Jin et al., 2009, 2012; Pornpitakpan, 2009; Yang, 2010; Dong, 2012). However, no framing effect was found in the emotion-focus condition. The current results also demonstrated that purchase motivation is one of the moderators of the option framing effect. Prospect theory holds that the option framing effect derives from the different sensitivities of customers to gain vs. loss and utility vs. money (Kahneman and Tversky, 1979; Hardie et al., 1993). The premise of this view is that purchase motivation stems from the gain in utility value of product. However, the motivations of customers are usually diverse (Ratneshwar et al., 1996; Higgins, 2002). The results of this study demonstrate that the purchase behaviors of customers are not affected by the different presentations of products, if only the feelings generated by the options are considered at the time of decision making. Thus, the existence of moderators of the option framing effect should remind managers and marketing institutions that the premise of this effect must be considered when they design sale strategies. If customers prefer the emotion induced by products to their utility and price, option framing will not take effect.

It is notable that the present results differ from the findings of Biswas (2009), in which the option framing effect was investigated in the purchase of automobiles in two processing modes, namely, rational versus experiential. Younger adults were instructed to make the optional product choice decisions in a strictly logical (rational) mode or in an emotional manner (experiential mode). As a result, the option framing effect was found in the experiential mode but not in the rational mode. This is contrary to the results of the present study. There are two possible explanations: first, the theoretical framework underlying the two studies is not identical. As previously mentioned the emotion-focus/information-focus condition emphasized more on the aims of decision making than the processing mode. As such, the emotion-focus condition is not similar to experiential mode and the information-focus condition is not similar to the rational mode. Second, the instructions in two studies were different, which may have also influenced participants’ decisions. In Biswas’s study, the instructions for the experiential mode were “A car purchase is supposed to be an emotional decision for most people. Therefore, please make your decisions in an emotional manner rather than one based on strict logic. That is, try to put your logical reasoning aside, and decide from an emotional point of view the options that you would want to add.” We are of the view that the terms *emotional manner* and *logical* may be ambiguous for participants. Whereas, the extent of pleasure induced by the options and the ratio of utility and price were explicit in the present study’s instructions, which may give participants some exact decision basis. Thus, different results were obtained in present study.

### Age and the Option Framing Effect

Age differences within the option framing effect were not found. All age groups had the same framing effect in the information-focus condition, whereas the framing effect failed to occur in the emotion-focus condition across all age groups. Consistent with our hypothesis, middle-aged adults had the same option framing effect as younger adults, which may due to their comparative cognitive abilities that the experiential mode depends upon. Unexpectedly, older adults did not exhibit more vulnerability to task presentation in comparison to younger and middle-aged adults. This finding is not consistent with our hypothesis that older adults would show a larger framing effect because of their reduced level of cognitive resources and intuitive processes. There are three possible explanations: the first is that older adults may weigh gains and losses differently than younger adults. In comparison to younger adults, older adults may give gains more weight because of their positive bias. The “gains seeking” co-exists with loss aversion in older adults. The gains seeking may produce a reverse option framing effect, meaning that more options are accepted under additive framing and fewer options deleted in subtractive framing. Therefore, a reverse option framing effect is linked to gains seeking reducing the existence of age differences in the option framing effect. This is supported by the results from research conducted into risky-choice framing. Using the monetary gambling task, Mikels and Reed (2009) discovered that when a decision task was personally relevant (participants received an amount of money proportional to their “winnings”), older adults were not influenced by framing and showed risk aversion in both framings. They attribute this result to the positive bias of older adults, which made them give less weight to losses than to gains. Thus, they did not show risk seeking in loss framing. The second reason is the confounding effect of older adults’ life experiences. Rönnlund et al. (2005) maintained that the life experiences of older adults compensated for their lesser cognitive resources, which explained why they did not show a larger framing effect than that of younger adults. Peters et al. (2011) reviewed the research on age differences among preference construction and concluded that preference was less constructed when older adults faced a familiar decision task. In the example of a daily shopping scenario, older adults were less likely to let the attractiveness of a discounted item influence their decision making, when its choice would require a larger minimum purchase than permitted by their usual budget (Tentori et al., 2001; Kim and Hasher, 2005; Kim et al., 2008). For Chinese older adults, there may be familiarity with shopping itself. Thus, they can stick to their shopping principles and not be influenced by framing. The third reason is the relatively lower level of cognitive demand of our framing task. In our task, participants can read the option list on a single page repeatedly and there was no time limit. Therefore, the demands on working memory and processing speed were not particularly high. Therefore, older adults did not construct more preferences than younger adults in the relatively simple option framing task.

### Age and Consumption Decisions

Although an age difference of framing effect was not found, two other results–the main effect of age and the interaction effect between age and focusing condition–were significant. Specifically, older adults tended to select more automobile options and accept higher prices than did younger and middle-aged adults, and this age difference was larger in the emotion-focus condition. In controlling for income, the main effect of age was still significant; the age difference of the accepted total price was not the result of the difference in income level among the three groups. Thus, we infer that the main effect of age may be related to the emotion regulation goal in older adults. Shafir (1993) notes that customers preferred “choosing” to “rejecting” because the former was a simpler task compared to the latter. Increased negative emotion may result in increased choice avoidance (Luce, 1998). Due to a greater focus on the emotion regulation goal, older adults tended to reject fewer options to avoid negative emotions in subtractive framing. For the same reason, they were inclined to select more options to optimize the emotional experience in additive framing. As for why the larger age differences were found in the emotion-focus condition, it mainly came from the decrease of the option numbers and prices accepted by younger adults in that condition. When younger adults were told to “only consider the degree of pleasure of the options,” it seems that they paradoxically devalued the options and accepted fewer options and prices. The exact reasons why younger adults were willing to accept great costs in information-focus condition are needed to be explored furtherly.

The data showed a slight but not significant difference between the two focusing conditions. Older adults paid slight more money in the emotion-focus condition than in the information-focus condition. This may be due to the car purchase task not being a self-related task for older adults. According to the selective engagement hypothesis (Hess, 2006), older adults show greater sensitivity to the self-related implications of a given task and they may engage more cognitive resources to do those high self-related task. It may therefore be inferred from this hypothesis that if the purchase task is highly self-related, such as health care plan for older adults, they may show a more obvious emotion regulated tendency.

In addition, participants in the middle-aged group accepted the same number and price of options in the two focus conditions. One reason for this result may be that utility, price and feelings about the products are all important to middle-aged adults. Such individuals still need to prepare for the future, such as preparing for retirement and ensuring long-term financial stability. Meanwhile, middle-aged adults begin to realize future limitations with increasing age and shift concerns to emotion satisfaction (Carstensen, 1992; Charles and Carstensen, 1999). Therefore, they may make the same decisions in the information- and emotion-focus conditions. The other reason may be that the total price of cars in our experimental materials was relative low for middle-aged group in comparison to their income. Though we controlled for the effect of income, the subjective values of options for individuals may still affect their decision making. If a customer regards a product as not particularly valuable, they may care less about how to select it. In order to explore whether social goals influence the purchase decisions of middle-aged adults, it would be of benefit to include products of higher subjective value into the research design.

### Limitations and Future Directions

The present study explored the role of motivations in the option framing effect among different age groups. The results imply that age characteristics must be considered in sales. It is suggested that the use of emotion in persuading older adults and the delivery of information to persuade younger adults may be effective.

The current study did have some limitations. First, the cognitive abilities of the three age groups were not directly measured. Though considerable literature reports a decline in cognitive ability as age increases, the absence of measurement of cognitive abilities limits the investigation of whether there is an interactive effect between motivations and cognitions in the option framing effect. Second, life experience may influence preference construction. Participants’ automobiles and general purchase experience should be objectively measured in future research. Thus enabling the three age groups with different purchase experience levels to be compared and the role of life experience in the option framing effect can be discussed further. Third, there was no control condition of focus. The absence of “baseline” condition makes it not clear if participants’ decision behavior in the information- or emotion-focus condition differs from typical behaviors for any age group. Finally, rating scores of the ratio of utility and price or the extent of pleasure of each choice could illustrate the desired options were higher in ratio of utility and price or extent of pleasure than undesired options. Thus, it may not directly examine the decision motivations of participants. Interviews or self-report capture of decision motivations from participants would be of benefit in future research.

## Author Contributions

HP designed the experiment and wrote the manuscript. SX and FR carried out experiments and analyzed experimental data. BP analyzed data.

## Conflict of Interest Statement

The authors declare that the research was conducted in the absence of any commercial or financial relationships that could be construed as a potential conflict of interest.
